# One Step Closer to the Understanding of the Relationship IDR-LCR-Structure

**DOI:** 10.3390/genes14091711

**Published:** 2023-08-28

**Authors:** Mariane Gonçalves-Kulik, Friederike Schmid, Miguel A. Andrade-Navarro

**Affiliations:** 1 Institute of Organismic and Molecular Evolution, Faculty of Biology, Johannes Gutenberg University of Mainz, 55128 Mainz, Germany; 2 Faculty of Physics, Johannes Gutenberg University of Mainz, 55128 Mainz, Germany

**Keywords:** intrinsically disordered regions, low complexity regions, protein structure, homorepeats, polyX, polyXY, AlphaFold

## Abstract

Intrinsically disordered regions (IDRs) in protein sequences are emerging as functionally important elements for interaction and regulation. While being generally flexible, we previously showed, by observation of experimentally obtained structures, that they contain regions of reduced sequence complexity that have an increased propensity to form structure. Here we expand the universe of cases taking advantage of structural predictions by AlphaFold. Our studies focus on low complexity regions (LCRs) found within IDRs, where these LCRs have only one or two residue types (polyX and polyXY, respectively). In addition to confirming previous observations that polyE and polyEK have a tendency towards helical structure, we find a similar tendency for other LCRs such as polyQ and polyER, most of them including charged residues. We analyzed the position of polyXY containing IDRs within proteins, which allowed us to show that polyAG and polyAK accumulate at the N-terminal, with the latter showing increased helical propensity at that location. Functional enrichment analysis of polyXY with helical propensity indicated functions requiring interaction with RNA and DNA. Our work adds evidence of the function of LCRs in interaction-dependent structuring of disordered regions, encouraging the development of tools for the prediction of their dynamic structural properties.

## 1. Introduction

Intrinsically Disordered Proteins or Regions (IDPs or IDRs) are full proteins or regions that lack a standard globular structural folding and present high flexibility [1]. They present a challenge in experimentally defining protein structures: in X-ray crystallography, these regions tend to be completely omitted, while in solution-state NMR, their signal is weak, even invisible. Alternative approaches are used to increase our knowledge about them, such as experimental analysis of the interactions between disordered and globular regions of proteins [2].

IDPs and IDRs are involved in a diverse range of biological processes, gene regulation and RNA processing, cell-cycle regulation, protein phosphorylation and other post-translational modifications, to indicate a few [3,4,5], with their flexible characteristics being one of the reasons for their functional advantages [6]. IDPs and IDRs also play a role in several diseases, such as cancer, diabetes, and cardiovascular and neurodegenerative diseases [7]. 

Studies of IDR amino acid sequences provided an overview of the reason why these regions are flexible. They present a high net charge and are mostly depleted of hydrophobic residues [8]. This often-reduced alphabet manifests as a bias in their amino acid composition, and as a result IDRs often overlap low complexity regions (LCRs). Some of these LCRs are simple tracts of repeated amino acids (homorepeats). Several diseases are associated with the expansion of poly-glutamine and poly-alanine homorepeats within IDRs [9,10].

In previous work, we studied the hypothesis that some of these LCRs in IDRs could provide them with a propensity to form a structure [11]. Although given the flexible nature of IDRs there is logically a reduced number of experimentally solved structures, using a few structures available for human proteins and homology we observed a high frequency of glutamic and glycine in LCRs with structural propensity; while most residues were unstructured, glutamic-lysine rich regions induced alpha-helical conformation, and we also observed less frequent beta-structures. Here, we aim to expand the results found in our previous work, taking advantage of predicted protein structures from AlphaFold (version v3), a recently developed deep learning tool for protein structure prediction [12]. 

In this study we are evaluating LCRs composed of a limited number of amino acids: homorepeats, here addressed as polyXs, and regions with only two types of amino acids, addressed as polyXYs. Previous studies targeted the extreme LCRs presented in our study, focusing on a functional [13] or evolutionary perspective [14] or compositional characteristics [15], but not on LCRs within IDRs. Others studied LCRs, comparing their structural information when associated or dissociated with IDRs [16], but not covering a high number of cases, again due to the low availability of experimental data.

Our aim is to expand the results already found in our previous work [11], where we used the alignment to PDB sequences to infer structure tendencies through homology. Here, we isolated the IDRs containing polyX and polyXYs to evaluate structure predictions generated by AlphaFold and analyze tendencies presented by distinct types of polyX and polyXY regions, seeking the expansion of our knowledge on the role performed by LCRs within IDRs and their structural relation.

## 2. Materials and Methods

We obtained 23,391.cif files from AlphaFold (v3) predictions [12] relative to the human reference proteome. Sequences longer than 2700 amino acids generate multiple models by sequence, so 3149 sequences with more than 1 model were removed from the set. We also removed AlphaFold sequences different from MobiDB sequences (see below), leaving a total of 20,034 AlphaFold models.

IDRs were downloaded from MobiDB version 4.1 [17], release 2021_11, and filtered according to the selected AlphaFold models, with 24,399 IDRs mapped in our dataset.

PolyXs were annotated using the web tool PolyX2 (version, URL, (accessed on 5 August 2022)) [18], specialized in the annotation of homorepeats in protein sequences. We considered homorepeat regions composed of only 1 type of residue in a window of at least 6 amino acids. PolyXYs were annotated using the web tool XYs [15], dedicated to the annotation of regions with at most 2 different residues in a specific window (each occurring at least twice). We limited the minimum region length to 6 amino acids. The minimum sizes of the search windows selected are the same as in our previous work, aiming to allow parallels between the two studies [11]. Only polyXs and polyXYs within IDRs were selected, with a total of 1913 and 9009 cases, respectively. 

Secondary structure annotations were generated with DSSP version 2.1.0 [19], following these designations: (H) for alpha-helix, (G) for 3/10 helix, (I) for pi-helix grouped as helices; (B) for beta-bridges and (E) for extended beta-strand ladders grouped as extended; (T) for turns, (S) for bends grouped as coils; and blanks (“ ”) for residues with low curvature in a not H-bonded structure designated as unmodeled regions. This DSSP version does not annotate polyprolines (PPII helices). Secondary structures for IDRs, polyX and polyXY regions, and the region of 100 amino acids surrounding the polyX/polyXY regions were extracted for analyses.

Two physicochemical properties of IDRs were calculated using localCIDER (version 0.1.18) [20]: Doolittle’s hydrophobicity and Net Charge Per Residue (NCPR). The latter is a metric developed by Pappu and co-workers that describes IDRs considering the position of the residues, nonpolar content and net charges with a score between −1 and 1 [21]. 

In-house scripts were developed in Python 3.8.10 to extract and transform the outputs from all data sources, with Biopython package (version 1.79) [22] used to extract DSSP (version 2.1.0) annotations [19]. All figures of protein molecular structures were produced with Chimera 1.15 [23]. Tables and statistical analyses were produced with R 4.2.2 and dplyr version 1.0.9, and figures with ggplot2 version 3.4.0. Gene Ontology enrichment was carried out with EnrichR, version 3.1 [24].

## 3. Results

We extracted all IDRs containing polyXs and polyXYs from sequences presenting a single prediction for the whole sequence in AlphaFold. A total of 1913 polyXs, with sizes ranging from 6 to 42 and mean size of 8.09 residues, and 9009 polyXYs, with sizes ranging from 6 to 63 and a mean of 7.69 residues were obtained (Supplementary Tables S1–S3). PolyXs were annotated within 1537 IDRs with sizes between 20 and 1036 (mean of 126.95) residues, with a mean of 1.24 and a maximum of 12 homorepeats per IDR. PolyXYs occur in 5853 IDRs, with sizes between 20 and 1985 (mean of 155.34) residues, and a mean of 1.54 and a maximum of 49 polyXYs per IDR. 

From the 20 canonical amino acids, 13 are present in our polyX set, mostly disorder promoters. Cysteine (C), isoleucine (I), leucine (L), phenylalanine (F), valine (V), tryptophan (W) and tyrosine (Y) are the missing residues, while asparagine (N) and methionine (M) are rare, with 1 homorepeat each (Table 1, Supplementary Table S3). The most common polyX is proline (P), closely followed by glutamic acid (E), with 461 and 452 cases, respectively. Serine (S) and glycine (G) follow, with almost half the frequency of the homorepeats mentioned above, with 283 and 244 cases, respectively. Serine (S) has the longest repeat, with 42 residues. According to AlphaFold predictions, polyPs are mostly covered by unfolded residues (99.3%), while half of polyQ regions are covered by helices (47.8% helices and 41.5% unfolded). Extended (beta) structures are uncommon amongst homorepeats, with no polyX with coverage above 1%.

**Figure 1:**
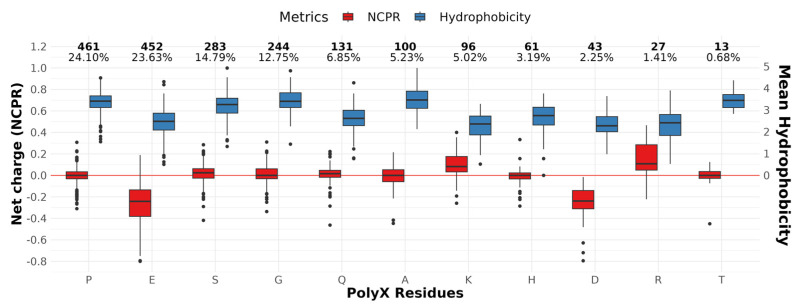
Canonical properties of IDR regions by polyX within the IDR. Left y-axis shows net charge fraction, while right y-axis shows average hydrophobicity with positive values only. The horizontal red line delimits uncharged IDRs. The numbers at the top of the figure show the total counts and percentages of each residue in our dataset. PolyM and polyN were omitted in this figure because only one case of each is available. IDRs were sorted by frequency.

We also evaluated some of the physicochemical properties that are important to understand protein foldability, with a focus on the IDRs containing polyXs. A low proportion of hydrophobic amino acids, which usually form the core of a folded protein, and a higher concentration of polar and charged residues, are properties common to IDR regions [8]. We evaluate in Figure 1 (see also Supplementary Table S1 for detailed values) how these properties are distributed in IDRs with different types of polyXs, considering NCPR as metric to evaluate net charges and Doolittle’s hydrophobicity for the complete IDR, including the repeated region. The IDRs containing charged polyXs show a lower mean distribution of hydrophobic residues, as expected; however, the IDRs containing homorepeats of two of the three polar uncharged residues on our top list, polyS and polyT, show hydrophobicity levels similar to those of IDRs for polyX of non-polar residues. Interestingly, repeats with charged residues are among the ones containing a higher coverage of helical structures in the polyX region; e.g., polyE, polyK and polyR. PolyD, despite being negatively charged and showing low hydrophobicity, does not show the same tendency for helicity. IDRs containing polyQ, also highly composed of helices in AlphaFold predictions, show a neutral charge content with a mean hydrophobicity similar to the observed in charged IDRs and lower than the remaining uncharged residues. NCPR behaves as expected, with negative charges for IDRs with negatively charged repeats and positive charges for positively charged ones. We can observe, however, that IDRs with polyX of positively charged residues show a greater tendency towards neutrality, which may suggest that the remaining residues of the IDR balance the global net charges. We do not observe this for IDRs with polyX of negatively charged residues, suggesting that there is a bias in IDRs towards being more permissive to negative charges, while local positive charges seem to require balancing. Since polynucleotides have negative charge due to the lateral phosphate groups, we hypothesize that this could be necessary to avoid unspecific interactions of IDRs with RNA and DNA.

A total of 128 different polyXYs were annotated (Supplementary Table S3). All 20 canonical amino acids were paired with different frequencies. The most present residues are proline (P), serine (S) and glycine (G), present in 19 different pairs, followed by aspartic acid (D), in 18 distinct pairs, and glutamic acid (E), present in 17 distinct pairs. The most uncommon residues were phenylalanine (F), tryptophan (W) and cysteine (C), with six, four, and three distinct pairs, respectively. The top 23 most common polyXYs cover almost 81% of our dataset. Of them, 15 different pairs are more than 90% covered by unfolded residues. From the remaining eight pairs with less than 90% coverage by unfolded residues, four show a greater preference for helices: polyER (76.3%), polyEK (36.8%), polyAE (27.6%), and polyKR (20.1%); and four are balanced between helices and coiled residues: polyDE (9.2% helices, 8% coil), polyEG (5.1% helices, 6.4% coil), and polyES (4.6% helices, 4.2% coil). Interestingly, glutamic acid is a common factor amongst polyXYs with higher helical content. 

PolyST and polyTV stand out for their higher coverage by extended structures, reaching almost 11% for 233 samples and 16.7% for 6 samples, respectively, compared to the common less than 1% coverage for most of the annotated pairs. Interestingly, all polyXYs containing more than 1% of extended residues are composed of threonine (T), which may suggest some tendency for AlphaFold prediction of extended residues when this amino acid is present. Further analyses are required due to the lower number of cases in the human proteome.

Extended regions were also not commonly detected in our previous work, showing extremely low counts in the PDB homology analysis [11]. Using the tool Local Structural Propensity Predictor (LS2P), which performs a statistical analysis of three-residue fragments extracted from SCOPe, polyEP showed some tendency to adopt extended structures in the polyXY region, but no polyEP region was predicted as extended by AlphaFold [11]. 

**Figure 2:**
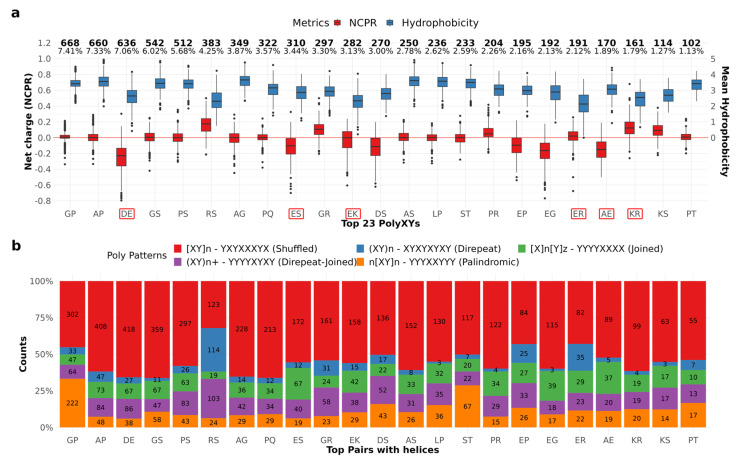
IDR properties and polyXY categorization. (**a**) Canonical properties of the IDR region by polyXY within the IDR. Left y-axis shows net charge fractions, while the right y-axis shows average hydrophobicity with positive values only. The horizontal red line delimits uncharged IDRs. The 23 more common pairs are shown, which represent almost 81% of the cases. The numbers at the top of the figure show the total counts and percentages of each residue in our dataset. IDRs were sorted by frequency and pairs with higher helical content are highlighted in red. (**b**) Categorization of polyXYs according to patterns observed in the polyXY region.

We performed the same physicochemical analysis described for IDRs with polyXs on IDRs with polyXYs. In Figure 2a (see also Supplementary Table S2 for detailed values) we can observe that the pairs composed of opposed charges, polyEK and polyER, show neutral charges, with lower mean hydrophobicity, which can also be observed for pairs within IDRs with unbalanced charges, polyDE, polyRS and polyKR in a smaller degree. Despite showing the netCharges and hydrophobicity levels expected in IDR regions, most of the pairs, highlighted in red, are between the types with a higher helical content in the polyXY region. Of those, only polyAE and polyES show hydrophobicity levels similar to pairs with low helix content. 

Mier and Andrade-Navarro [15] proposed a taxonomy to explore the diversity of polyXYs, based on the organization pattern of the two residues present in the polyXY. Three classes were defined: Direpeats are formed by periodical XYs; Joined are the ones where a number of X is followed by a number of Ys; and Shuffled are the ones not following the previous categories. We characterize our polyXYs with this taxonomy and add two extra layers to the shuffled category: Direpeat-Joined being the polyXYs composed by a homorepeat and a direpeat; and Palindromic being the ones with a palindromic pattern. We can observe in Figure 2b (see also Supplementary Table S2) that polyGP and polyST show a high frequency of palindromic polyXYs, while polyRS show high frequency of Direpeat-Joined and Direpeat patterns. Further investigation is required to evaluate if and how these patterns affect the 2D structures surrounding these polyXY regions.

## 4. Discussion

### 4.1. AlphaFold Predictions Support Previously Reported LCR Preferences for Structured Residues

**Figure 3:**
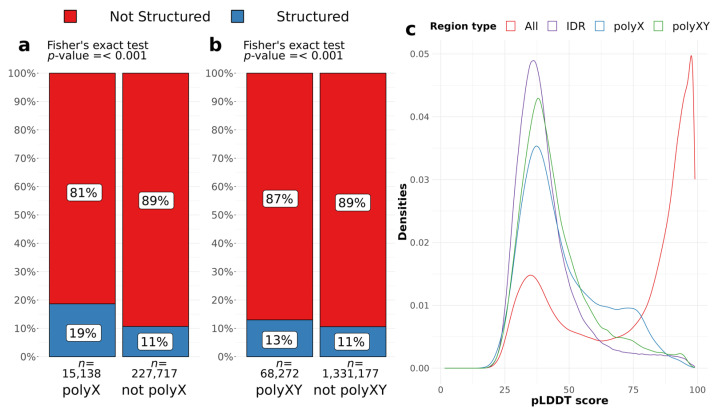
Evaluation of residues of polyX and polyXY in IDRs covered by secondary structures. (**a**,**b**) Proportion of the residues of an LCR type found in IDRs (polyX and polyXY, respectively), covered by structured (blue) and unstructured (red) residues. A significantly higher propensity for a LCR region to be structured than other residues from the IDR can be observed. Only IDRs with LCRs were considered for this statistical analysis. (**c**) Densities of pLDDT scores for all residues, IDR regions, and LCRs of two types (polyX and polyXY) within IDRs.

To assess whether polyX and polyXY regions can induce structural gain in IDR regions, we evaluated AlphaFold structure predictions for all IDRs, considering the presence of a polyX/XY, and the annotation of a secondary structure by AlphaFold. We accepted all structures predicted by DSSP, considering blanks, which are the residues with low curvature in a not H-bonded structure, as unstructured (see Methods for details). Around 6% of all residues of IDRs are polyXs and 19% of those are structured, while 11% of the remaining IDR residues are structured (Figure 3a). PolyXYs cover almost 5% of the IDR residues and are less structured, presenting 13% of coverage compared to 11% of the structured residues outside polyXY regions (Figure 3b). PolyXs are more likely to contain a structured residue than the surrounding IDR [*p* < 0.001, Odds ratio = 1.92, 95% CI = 1.84–2.00]. PolyXYs, despite the lower effect size, still have odds of being structured 1.26 times more than IDR residues outside polyXYs [*p* < 0.001, Odds ratio = 1.26, 95% CI = 1.23–1.29] (Figure 3b). Fisher’s exact test was used for both sets.

Along with structure predictions, AlphaFold provides the per-residue confidence score (pLDDT) on a scale between 0 and 100, which indicates the tool’s confidence in the prediction of the structure adopted by the residue. Scores with values below 50 are considered a prediction of disorder [12]. The evaluation of pLDDTs can also support the findings discussed above, where regions containing polyX/XYs show a higher pLDDT compared to complete IDR regions. Figure 3c shows the distributions of pLDDT scores for residues in IDRs, polyXs and polyXYs, as well as for the full set of residues as a benchmark. We can observe that polyX followed by polyXYs shows a lower peak of scores between 25 and 50 and an increase to the right tail between 50 and 75. The significance of the difference between distributions was confirmed through Wilcoxon rank test, with *p* value < 0.001, indicating that IDR scores are significantly lower than polyX and polyXY scores.

We can also observe in Supplementary Tables S1 and S2 that not all predicted IDRs extracted from MobiDB present pLDDT scores lower than 50, achieving a median value as high as 97.1 for IDRs containing polyX and 98.0 for polyXYs. In fact, the pLDDT score has shown competitive performance compared to the state-of-the-art disorder predictors [25]. However, these prediction tools do not always agree on their confidence scores and covered residues, so these cases should be taken with caution.

Taken together, these results reinforce the findings of our previous work [11], where we could observe a preference for regions covered by polyX and polyXYs in adopting secondary structure compared to the other IDR residues, with polyX having stronger propensity for structure than polyXY.

### 4.2. Some polyX and polyXYs Show Distinct Helical Propensity Depending on Their Position in the Sequence

**Figure 4:**
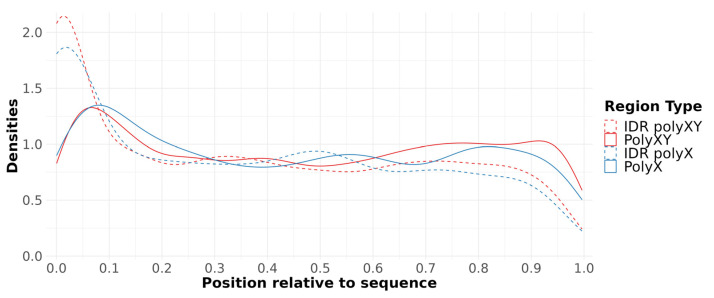
Position of polyX/XY relative to protein sequence. IDR distributions are presented for comparison.

As discussed by Delucchi et al. [10], homorepeats in IDRs present a propensity for accumulation at the protein sequence N-terminal. Our data show the same distribution, with a higher concentration at the N-terminal and smaller at the C-terminal not only for polyXs, but also for polyXYs (Figure 4).

To better understand helix propensities in polyXs along the sequences, we studied the properties of the repeats (entirely) located at the termini of the protein sequence (defined as the N- or C-terminal 100 amino acids of the protein, or the 30% terminal sequence if the protein has fewer than 300 amino acids (Table 1). As expected, the distribution of repeats follows the N-terminal preference observed in Figure 2; however, the helical coverage does not follow the same trend, being distributed homogeneously. Among polyXs with higher helical content, polyQ and polyE show slightly greater coverage towards the C-terminal, regardless of lower repeat counts in this region. PolyA, on the other hand, presents greater depletion of helix content at the N-terminal, the region with the highest frequency of these repeats. We can also notice that polyK has the lowest N-terminal content and helical content depletion in the non-termini region when compared to polyXs with higher helical content described above.

The same analysis was performed on polyXYs and is detailed in Supplementary Table S4. PolyAG shows a higher accumulation at the N-terminal of the sequence, while polyDE shows the inverse preference. However, no specific structural preferences can be observed considering the helical content of the polyXYs. Among the polyXY with higher helix coverage, polyES and polyEK are less concentrated at the N-terminal of the sequence and accumulated at non-termini regions. PolyER shows high content of helices in non-termini and C-terminal, while polyAK is more concentrated at both termini regions, with higher coverage of helices at the N-terminal. These results suggest that while both polyX and polyXY behave similarly regarding some accumulation at N-terminal, polyXY are more heterogeneous in respect to the position of helical structures while polyX with high helical content were more distributed. 

### 4.3. Structural Context of polyX in IDRs

**Figure 5:**
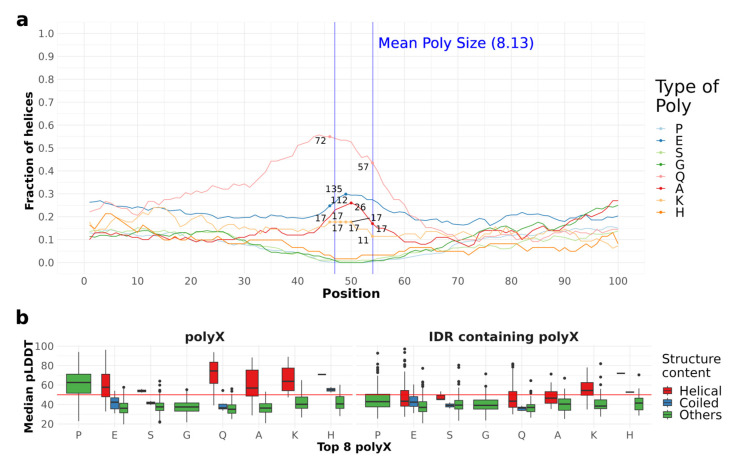
Fraction of helical residues and pLDDT distribution for polyX. (**a**) The fraction of helical residues for the eight most common polyXs is presented as lines in the plot, whilst just the higher and smaller counts within the region are shown per polyX type, to delimit the region counts compared to the fractions. The blue vertical lines delimit the mean size of polyX regions for reference. (**b**) Distribution of pLDDTs for polyX and IDRs grouped by type of secondary structure content. Helical indicates polyX with at least four consecutive residues predicted to be in helical conformation. Coiled indicates polyX with at least four consecutive residues predicted as coiled.

We obtained the secondary structure annotations from the AlphaFold (v3) predictions using DSSP (2.1.0) and extracted 100 residues surrounding the polyXs and polyXYs, with the repeat at the center of the region. Figure 5a presents the counts of helical residues for the eight more common polyXs. Glutamic-acid (E) shows the highest counts within the homorepeat region, covering around one third of the sequences composed of this residue in our set. PolyQ follows, reaching half of the sequences composed of glutamine homorepeats. In agreement with the findings of Totzeck et al. for polyQ in general [26], we also observe a preference for helices to be at the N-terminal of polyQ in IDRs.

**Figure 6:**
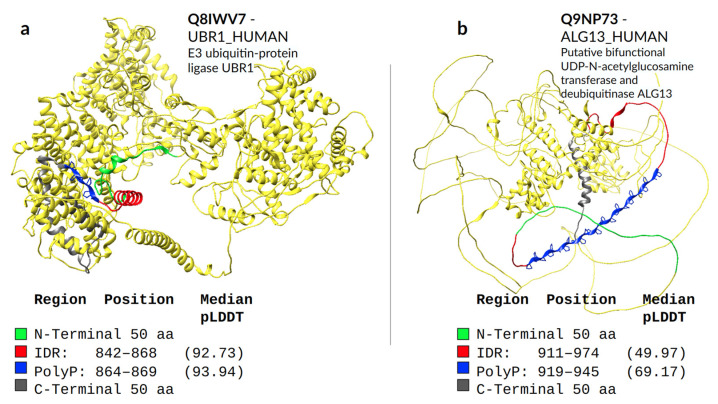
AlphaFold structural predictions for proteins with polyP containing IDRs. The polyP is represented in blue, including the side chain represented with sticks. (**a**) Predicted structure for protein Q8IWV7-UBR1_HUMAN-E3 ubiquitin-protein ligase UBR1, which presents the highest pLDDT we observed for a polyP in an IDR and for the corresponding IDR. (**b**) Predicted structure for protein Q9NP73-ALG13_HUMAN-Putative bifunctional UDP-N-acetylglucosamine transferase and deubiquitinase ALG13. This protein presents the longest polyP in our dataset, with 27 residues.

We used pLDDT scores to assess the confidence levels of AlphaFold predictions for secondary structure. We defined polyXs as helical if they had at least four consecutive residues predicted to have helical conformation, which would indicate at least one turn covering the homorepeat. PolyX were defined as coiled if four consecutive residues were predicted to have coil conformation and all other polyX were grouped as “Others”. Residues in extended conformation in polyX in IDRs were extremely infrequent (nine residues in total) and were not considered. We represent in Figure 5b the distribution of pLDDT values for each polyX type and for the IDRs containing them. Helical polyXs show average scores higher than 50, while coil and unfolded follow mostly the global IDR scores. Interestingly, although most polyPs are not helical or coiled, they have high confidence scores. This is due to them being predicted as polyproline helices (see two examples in Figure 6). This characteristic, however, is just noticeable through visual inspection of the structure, since the DSSP version used in this work does not annotate PPIIs. IDRs present lower scores than the corresponding polyX, confirming the higher structural propensity of polyX within IDRs.

**Figure 7:**
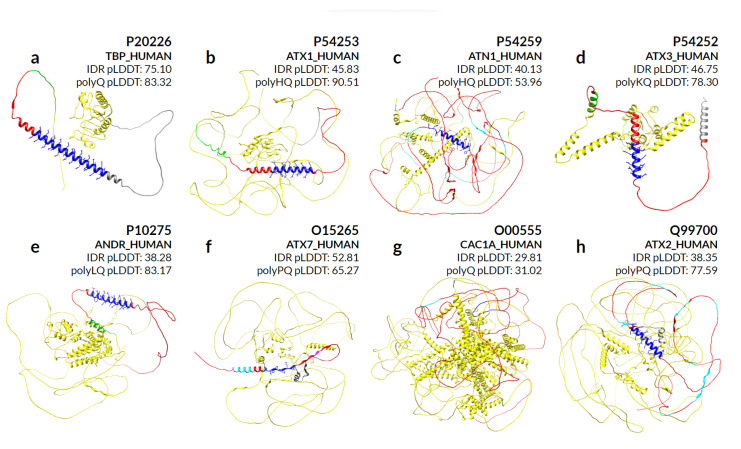
AlphaFold structural predictions of proteins associated with diseases caused by polyQ expansion. PolyQ/QX are shown with stick representation in blue. Green and gray residues highlight N and C-terminal 50 amino acids from the center of the polyQ/QX, respectively. Residues in red delimit the IDR region and cyan, when shown, represents other polyXYs within the polyQ containing IDR. Median pLDDT values are shown for the IDR and polyQX regions (blue), while the remaining values are available in Supplementary Tables S1 and S2. The following proteins are illustrated: (**a**) P20226-TBP_HUMAN; (**b**) P54253-ATXN1_HUMAN; (**c**) P54259-ATN1_HUMAN; (**d**) P54252-ATXN3_HUMAN; (**e**) P10275-ANDR_HUMAN; and (**f**) O15265-ATXN7_HUMAN. Extra purple and magenta colors delimit adjacent polyXYs (polyAR, polyAG, polyPQ and polyPR); (**g**) O00555-CAC1A_HUMAN; (**h**) Q99700-ATX2_HUMAN.

We make a special mention of the nine proteins for which abnormal expansion of polyQ is responsible for genetically inherited neurodegenerative diseases that result in protein aggregates in the brain [27]. By evaluating the secondary structures predicted by AlphaFold in eight of these proteins (Figure 7), we can observe that different sequence compositions lead to helical preference in almost all cases. P42858 (HD) is not part of our evaluation due to the large size of the sequence.

The protein P20226 (TBP_HUMAN) in Figure 7a contains only one long polyQ, with 38 residues covered by helices. Proteins P54253 (ATX1_HUMAN) in Figure 7b and P54259 (ATN1_HUMAN) in Figure 7c share a polyHQ, the first with a more scrambled pattern also completely covered by helices, while the second is with a clear insertion of polyQ within polyH and most of the helical content covering only the polyQ. Proteins P54252 (ATXN3_HUMAN) in Figure 7d and P10275 (ANDR_HUMAN) in Figure 7e are composed of a polyKQ and a polyLQ, respectively, where these residues seem to contribute to the helicity of the region that extends beyond the polyXY in both directions.

For the O15265 (ATXN7_HUMAN) protein in Figure 7f, the surrounding polyQ region shows a more diverse set of small overlapping polyXYs. In order: polyAR, polyAG, polyPQ and polyPR, with its helical content actually preceding the polyPQ region. Its polyQ content is in fact the lowest of all polyQ toxicity proteins, with four contiguous and two non-contiguous residues, which may explain its inability to maintain the helical structure formed by the previous polyXYs. Protein O00555 (CAC1A_HUMAN) in Figure 7g is the only example that shows no helical content in the AlphaFold prediction, despite showing a polyQ stretch of 13 residues following 2 prolines. Finally, Figure 7h represents protein Q99700 (ATX2_HUMAN), which is analyzed in detail in the next section.

We can also observe IDR pLDDTs compared to polyQ and polyQX for all protein representations in Figure 7. The protein P20226 shows a high pLDDT score for both regions, indicating a divergence amongst several IDR prediction methods on whether the region is disordered. On the other extreme, proteins P54253 and P10275 show a much higher confidence in the IDR region, with values of 90.51 and 83.17, respectively.

### 4.4. PolyXs Can Help in Understanding polyXY Structural Patterns

Interestingly, as illustrated above in the proteins with polyQ expansions, there are a number of polyX that overlap polyXY in IDRs. Given that we were able to characterize the structural properties of some of the most frequent polyXs, and that polyX has higher structural propensity than polyXY, in this section we study polyXY structural propensities by their overlap with polyX.

**Figure 8:**
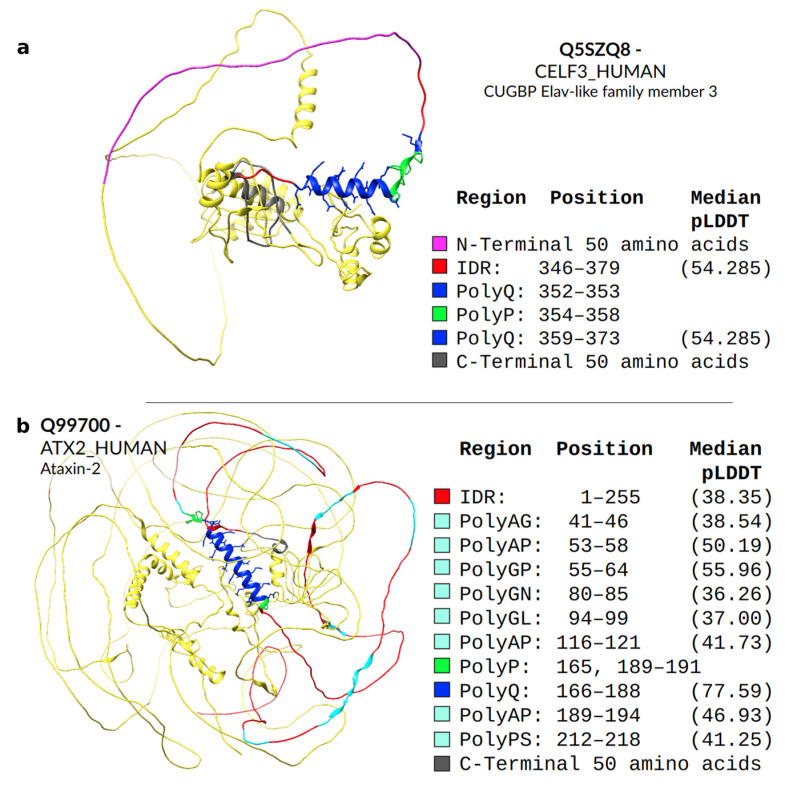
AlphaFold structural predictions for two proteins with a polyP overlapping a polyPQ within an IDR. Stick representation was used for the polyQP, with the prolines colored in green and the glutamines colored in blue (**a**) Predicted structure for Q5SZQ8-CELF3_HUMAN-CUGBP Elav-like. (**b**) Prediction structure for Q99700-ATX2_HUMAN-Ataxin-2 with several other polyXY highlighted in cyan.

We crossed the two datasets to evaluate if some structural patterns are detectable in these overlaps. A total of 636 polyXs are within polyXYs (33.2% of polyXs). Table 2 describes how each polyX overlaps polyXYs. PolyQ is again the most common homorepeat, with more than 42% of the polyXs within polyXYs, and with more than 51% of them with at least one turn of a helix over the repeated region. This overlap is, however, spread among 10 of the 15 observed polyXY pairs that include glutamine, with polyPQ being the most common, with a total of 19 cases, and only two of them containing helices. In fact, these two polyPQs with helical content, Q5SZQ8-CELF3_HUMAN-CUGBP Elav-like family member 3 (QQPPPPPQQQQQQQQQQQQQQQ) and Q99700-ATX2_HUMAN-Ataxin-2 (PQQQQQQQQQQQQQQQQQQQQQQQPPP), are the ones with the longest contiguous polyQ stretch amongst polyPQs (15 and 23 Q residues, respectively) and highest median pLDDT values (Supplementary Tables S1 and S2). This observation may suggest an effect similar to that proposed by Urbanek et al. [28], where polyP exerts a conformational perturbation on the helical segment of polyQ. We can observe this trend in both predicted structures (Figure 8). Protein CUGBP Elav-like, in Figure 8a, shows a break in the helix right after the end of the polyQ stretch, while ATX2 in Figure 8b has several short polyXYs, mostly unstructured and composed of polyPX along the IDR region, colored in cyan, and a helical polyPQ region. We note that these two proteins also belong to the set of overlaps of polyP with polyPQ, and there are another 37 cases, all of which present only unstructured residues.

Another relevant polyXY, polyDE emerged from overlaps with polyXs. It is the most frequent polyXY, with almost 47% of the cases of polyE and more than 85% of the polyD within polyXYs, totaling 75 cases. PolyDs within polyXYs are in general rare, with a total of 14 unstructured cases; however, polyE, the second most common in our set of homorepeats, has more than 46% of occurrences within polyDE, with almost a third of them containing helices. As polyEs are the polyXY most covered by helices within IDRs, further analyses are required to better understand the differences between the two conformations for these extremely acidic regions. 

### 4.5. Structural Context of polyXYs with Helical Propensity in IDRs

**Figure 9:**
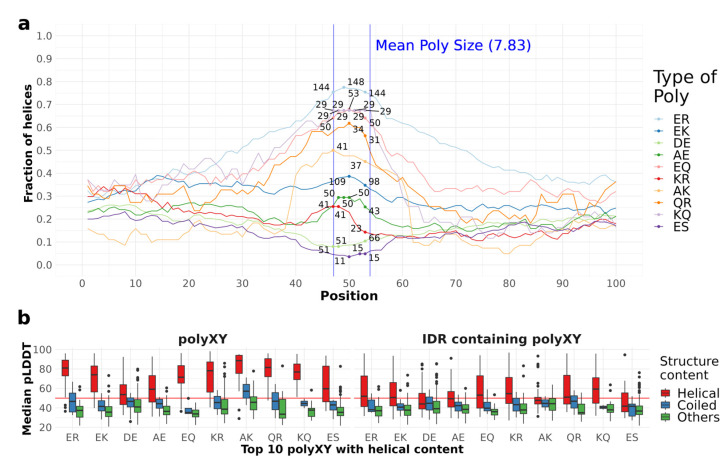
Fraction of helical residues and pLDDT distribution for polyXYs. (**a**) The fraction of helical residues for the 10 most common polyXYs is presented as lines in the plot, whilst just the higher and smaller counts within the region are shown per polyX type, to delimit the region counts compared to the fractions. The blue vertical lines delimit the mean size of polyXY regions for reference. (**b**) Distribution of pLDDTs for polyXY and IDRs grouped by type of secondary structure content (defined as in Figure 5).

In this section, we apply to the top 10 polyXY with the highest helical content the same analyses we used to study the structural context of polyX in Section 4.3. First, we analyze the amount of helical structure in the 100 residues surrounding each polyXY type (Figure 9a). The 10 polyXYs showed counts between 11 and 148 within the polyXY region.

PolyER presents a higher frequency of helices on the polyXY region in more than 70% of the sequences, reaching 148 cases with helical conformation at the same aligned position. Interestingly, polyR is an uncommon polyX, with a total of 27 occurrences in human IDRs, reaching around 30% of helix coverage; however, for polyER the bias towards helicity increases. PolyER did not present the same high propensity for helicity in our previous work [11].

PolyEK show lower helix frequency (around 40%), not only on the polyXY region but with similar counts through all the 100 residues surrounding the region. These findings confirm again the propensity found in our previous study [11]. Interestingly, polyE has the second highest helical coverage in the repeated region, while polyK by itself has a lower propensity (Figure 5a). PolyAK, differently, shows a peak with 37 to 40 cases with helical content in the polyXY region and a pronounced drop outside. PolyXYs containing glutamine (Q) also show helical propensity when associated with glutamic, arginine and lysine, but their occurrence in IDRs is less common than that of polyXY pairs containing glutamic (E).

When comparing the pLDDT values on IDRs and their equivalent polyXYs (classified by their secondary structure content in helical, coiled and others, as carried out for polyX in Section 4.3), we observe the same trend as in the polyX dataset, with median values above 50 in polyXYs and below 50 for most IDR regions (Figure 9b). Again, very few residues were found in extended conformation (six residues in total). PolyDE is the polyXY with the lowest prediction confidence for polyXY with helices by AlphaFold, while polyAK and polyER show the highest confidence. 

Taken together, the results of the analyses presented in this section indicate that the helical propensities of polyXY have not much to do with the propensities of the corresponding polyX and polyY, and point to polyER as a frequent LCR with the highest helical propensity. 

### 4.6. Proteins Containing polyXYs in IDRs Are Enriched in Functions Requiring RNA and DNA Binding

To find out if polyXY with high helical propensity has an associated functional role in the human proteome, we performed a Gene Ontology (GO) enrichment analysis focused on the top 10 polyXYs with the highest helical coverage in the polyXY region (maximum cutoff value of 0.05 for the adjusted *p* value; Supplementary Table S5). Comparing the three aspects considered in GO, biological processes gave the highest number of enriched terms; since the other two aspects, molecular function and cell component, gave related terms, we will focus our analysis on the biological processes component in the next paragraphs. 

**Figure 10:**
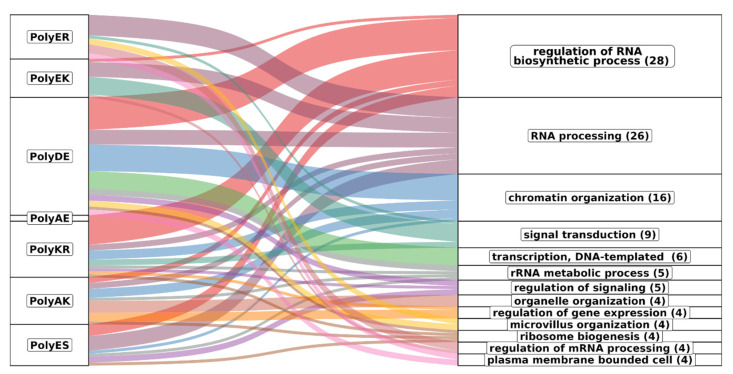
Functional associations of polyXYs with high helical propensity. Associations of biological process GO terms (significant adjusted *p* value < 0.05) with polyXYs with high helical propensity in IDRs. The GO terms are grouped and sorted by common parent term, with different colors associating the GO terms with polyXYs. Values in parentheses show how many GO terms were annotated for each parent term.

Figure 10 represents the associations of biological process GO terms with proteins with helical rich polyXY in IDRs. Of the top 10 polyXY, 7 had enriched terms. In the figure, polyXYs are ordered by most helical content, while the biological processes are ordered by number of enriched terms. Parent terms with at least four child terms are shown, covering 70% of all annotated terms.

The main result is that the terms are mostly related to protein functions that require interaction with RNA and DNA (see for comparison the results for polyX in Supplementary Table S6). Regulation of RNA process is spread through almost all polyXYs, being uncommon in polyEK, absent in polyER and the only biological process enriched for polyAE. RNA processing is more evenly disseminated in all enriched polyXYs. PolyER and polyEK show the lowest variation of function after polyAE. When observing the 11 remaining biological processes, polyER is only enriched on 4 of them, signal transduction, microvillus organization, regulation of mRNA processing and plasma membrane bounded cell. PolyEK is enriched in two of them, signal transduction and regulation of mRNA processing.

**Figure 11:**
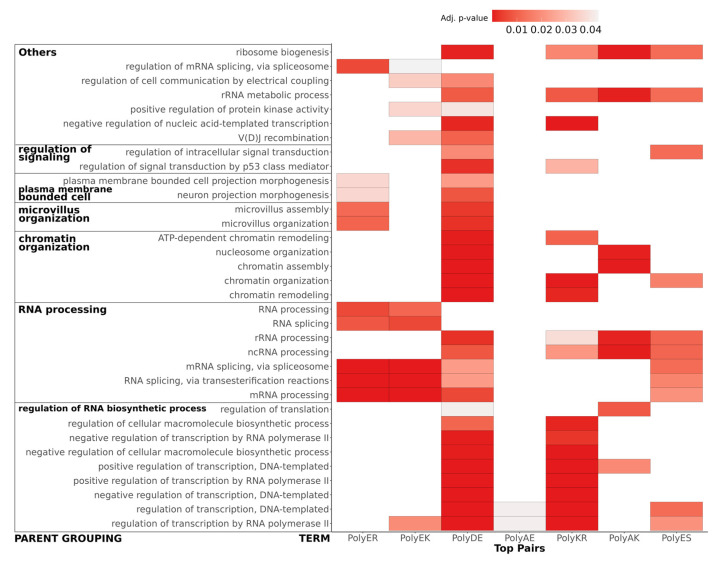
Co-occurrence of enriched biological process GO terms between polyXYs with high helical propensity. Heatmap of GO terms enriched in more than one of the top 10 polyXY with higher helical content in the polyXY region, where darker red represents more significant adjusted *p* values up to 0.05. Common parent terms are used to group terms and, when available, are shown on the left.

In Figure 11 we represent separately the association of enriched GO terms for biological processes that appeared associated with at least two different types of polyXY. A total of 34 co-occurrences were found, with proteins with polyDE presenting a higher enrichment on most terms.

PolyAE proteins show the worst adjusted *p* value for both enriched terms. PolyKR proteins share most of the biological processes of regulation of RNA biosynthetic process with polyDE, missing only regulation of translation, which is enriched in polyAK proteins instead. PolyER and polyEK are enriched in most of the RNA processing terms but are not involved in rRNA processing and ncRNA processing, enriched in polyES. 

Although these polyXYs were selected for their helical propensity, not all the polyXYs of the category have helical content. We evaluated if the proteins responsible for the enrichment contained helical polyXY or not (Supplementary Table S5). We note that considering polyER-, polyEK- and polyES-enriched terms related to RNA processing, we can observe that ncRNA and rRNA processes were enriched for polyES without helical content, while all remaining processes for these three polyXYs show some level of association with helical content, markedly in polyER and polyEK. We take this result as a suggestion that the helical propensity of these polyXYs can play a role in these biological functions. More investigations are required to support this hypothesis.

## 5. Conclusions

IDRs initially considered unstructured are increasingly taken as regions with diverse conformational preferences and high flexibility, which, in fact, bring essential advantages for the living organism to adapt and survive [1].

Our purpose was to address two simplified aspects of these regions, structure and low complexity, and better understand their relationship: are we able to identify patterns in secondary structure preferences within IDRs based on their regions with the lowest sequence diversity content? Before answering this question, we should evaluate why the propensity to adopt a secondary structure before contacting a partner molecule has advantages. The binding process described as conformational selection suggests that pre-folded conformations can present a higher affinity in the binding process than their flexible counterparts when binding competent interacting partners are available. It is worth mentioning that the secondary structure propensities are not the determining factor showing that this mechanism occurs, since stronger interactions may overcome the structure predisposition. Regardless, a better understanding of secondary structure propensities may clarify when these processes, less common for IDR binding, may occur [29,30,31,32].

Answering now the question of whether polyXs and polyXYs can induce structure in IDR regions, our findings support this theory. We could establish a parallel between AlphaFold predictions and our previous study based on proteins with known structures deposited in the PDB and their homologs, for both polyXs and polyXYs [11]. In fact, not only the helical content of distinct kinds of LCRs was confirmed, but also the AlphaFold’s confidence scores of regions containing those LCRs are higher.

From the homorepeat perspective, comparing this study using AlphaFold predictions to our previous one using PDB structures, polyE presented the same tendencies in adopting helical structure, and a new polyX emerged with similar tendencies, polyQ. In the previous study, this homorepeat presented extremely low case counts due to a lack of homologous PDB structures, while the increase in structures provided by AlphaFold predictions allowed the surfacing of its helical propensities, which agrees with experimental observations [28].

The same effect was observed in polyXY analyses. PolyER emerged with a preference to adopt helices, while polyEK remained as one of the polyXY with higher helical tendencies. The new set of polyXYs shows that, in fact, the association of charged residues can play a role in these propensities.

All of the polyX and polyXY types that showed higher helical content also present a high number of unstructured residues, suggesting that a more complex pattern within each kind of LCR might exist and requires further investigation.

From a broad and more investigative perspective, we evaluated the distribution of these LCRs throughout their sequences. Some interesting patterns emerged, such as polyAG concentration at the N-terminal of the sequence, without a particular preference for those with helical content, and polyAK concentration at the N-terminal with a higher prevalence of helical content.

Ultimately, we searched for the biological function of the different types of polyXYs with a propensity for helices, observing a strong association with RNA and DNA binding-related functions, with polyER and polyEK showing a strong association with RNA processing for both helical and unstructured polyXY. We take this as an indication that, differently to polyX, polyXYs of one or two charged amino acids are generally exploited for interactions with polynucleotide chains, possibly realizing conformational changes, mostly to helical structures if secondary structure is involved. This result agrees with the growing body of evidence reporting the modes by which IDRs interact with RNA and DNA [33,34,35].

The complexity of understanding IDRs can now be better approached, thanks to AlphaFold; however, our results should be taken with caution, as AlphaFold’s predictions could be biased if its training set had been enriched in structures with polyX and polyXY located in particular structuring environments. In general, we take the consistency of AlphaFold’s predictions with our previous results and with other well-known structural facts about LCRs (for example, the helical propensity of polyQ) as a good indicator of the usefulness of its predictions in IDRs. In any case, the prediction of the structures of IDRs is still problematic and must be analyzed with caution, in a synergy between computational and experimental techniques [36]. Our findings suggest that the establishment of local structure within IDRs could be fundamentally related to the content of LCRs present in those regions. Further steps in cataloging these LCRs, such as additional AlphaFold modeling of complete and local sequences without the incorporation of templates, and their comparison with complementary modeling techniques, are among the work necessary to support future findings on mechanisms of biological functions and interactions. 

## Figures and Tables

**Table 1:** Counts and percentage of each type of polyX residue by position in the sequence (see definition in Section 4.2), with average coverage by helices.

	Total		N-Terminal		Non-Termini		C-Terminal	
Res	Count	Helix/%	Count/%	Helix /%	Count/%	Helix/%	Count/%	Helix /%
Total	1913	18.18	455 (37.81)	15.11	1153 (48.75)	14.25	305 (13.43)	10.95
P	461	0.03	83 (18.00)	0.17	315 (68.33)	0.00	63 (13.67)	0.00
E	452	28.49	79 (17.48)	25.05	303 (67.04)	28.96	70 (15.49)	30.38
S	283	0.94	53 (18.73)	1.57	183 (64.66)	0.74	47 (16.61)	0.96
G	244	0.12	112 (45.90)	0.26	99 (40.57)	0.00	33 (13.52)	0.00
Q	131	47.79	30 (22.90)	37.42	87 (66.41)	49.79	14 (10.69)	57.64
A	100	22.28	54 (54.00)	12.68	33 (33.00)	34.56	13 (13.00)	31.01
K	96	15.71	8 (8.33)	25.00	47 (48.96)	9.42	41 (42.71)	21.11
H	61	2.25	12 (19.67)	0.00	39 (63.93)	3.53	10 (16.39)	0.00
D	43	2.23	5 (11.63)	1.43	24 (55.81)	3.00	14 (32.56)	1.19
R	27	29.63	14 (51.85)	7.14	13 (48.15)	53.85	0 (0.00)	0.00
T	13	1.10	3 (23.08)	0.00	10 (76.92)	1.43	0 (0.00)	0.00
M	1	85.71	1 (100.00)	85.71	0 (0.00)	0.00	0 (0.00)	0.00
N	1	0.00	1 (100.00)	0.00	0 (0.00)	0.00	0 (0.00)	0.00

**Table 2:** Description of polyX overlap with polyXY, with counts for the most common polyXYs and their helical content.

Res	X in XY	Perc.	Helix Coverage	N in Distinct XY^1^	Most Common XY	Helix on Common XY
P	153	33.19%	0 (0.00%)	12 in 19	LP (50–32.68%)	0 (0.00%)
E	135	29.87%	44 (32.59%)	12 in 17	DE (63–46.67%)	20 (45.45%)
S	95	33.57%	1 (1.05%)	12 in 19	PS (20–21.05%)	0 (0.00%)
G	92	37.70%	0 (0.00%)	12 in 19	GS (39–42.39%)	0 (0.00%)
Q	56	42.75%	29 (51.79%)	10 in 15	PQ (19–33.93%)	2 (6.90%)
A	35	35.00%	11 (31.43%)	9 in 14	AG (10–28.57%)	3 (27.27%)
H	24	39.34%	1 (4.17%)	8 in 14	HP (8–33.33%)	0 (0.00%)
K	19	19.79%	2 (10.53%)	9 in 15	EK (4–21.05%)	1 (50.00%)
D	14	32.56%	0 (0.00%)	2 in 18	DE (12–85.71%)	0 (0.00%)
R	9	33.33%	5 (55.56%)	7 in 15	PR (2–22.22%)	1 (20.00%)
T	3	23.08%	0 (0.00%)	3 in 15	GT (1–33.33%)	0 (0.00%)
M	1	100.00%	1 (100.00%)	1 in 9	KM (1–100.00%)	1 (100.00%)

1 Number of polyXs in all available polyXYs containing the residue (e.g., polyP is present in 12 different polyXYs out of 19 different polyXYs containing proline).

## Data Availability

The data presented in this study are available on request.

## Supplementary Materials

The following supporting information can be downloaded at: https://www.mdpi.com/article/10.3390/genes14091711/s1, Table S1: Properties of polyX within IDRs with AlphaFold structural predictions; Table S2: Properties of polyXY within IDRs with AlphaFold structural predictions; Table S3: Summary of polyXs and polyXYs and distribution of helical content; Table S4: Counts of polyXYs by sequence region and distribution of helical content; Table S5: Enriched GO terms by polyXY with high helical propensity; Table S6: Enriched GO terms by polyX.
