# Supplementary material for: One Step Closer to the Understanding of the Relationship IDR-LCR-Structure
Source: Genes (Basel). 2023 Aug 28;14(9):1711. doi: 10.3390/genes14091711 (PMC10531472; doi:10.3390/genes14091711)
Supplement: Supplementary file 1 [file genes-14-01711-s001.zip › supp_materials.pdf]

Supplementary tables

Supplementary table S1: Properties of polyX within IDRs with AlphaFold structural predictions. Columns indicate: UniProt identifier (seq name), identifier of the polyX (poly name), poly size, start and end of the polyX in the query sequence (poly seq start and poly seq end), amino acid type, identifier of the IDR (idr name), size of the IDR (idr size), start and end of the IDR in the query sequence (idr start and idr end), idr mean hydrophobicity, idr mean ncpr, sequence of the polyX (poly aa), secondary structure from DSSP in the polyX including blanks (poly ss), detailed list of pLDDT scores for the polyX region (poly plddt), poly median plddt, sequence of the IDR (idr aa), secondary structure from DSSP in IDR including blanks (idr ss), detailed list of pLDDT scores for the IDR region (idr plddt), idr median plddt, 50 amino acid sequence to the left and to the right of the polyX center (poly seq left and poly seq right) and their secondary structure annotations (poly ss left, poly ss right). Pipe signs ("|||") indicate the end of the sequence range.

Supplementary table S2: Properties of polyXY within IDRs with AlphaFold structural predictions. Columns indicate: UniProt identifier (seq name), identifier of the polyXY (poly name), poly size, start and end of the polyXY in the query sequence (poly seq start and poly seq end), amino acid XY, topology XY, identifier of the IDR (idr name), size of the IDR (idr size), start and end of the IDR in the query sequence (idr start and idr end), idr mean hydrophobicity, idr mean ncpr, sequence of the polyXY (poly aa), secondary structure from DSSP in the polyXY including blanks (poly ss), detailed list of pLDDT scores for the polyXY region (poly plddt), poly median plddt, sequence of the IDR (idr aa), secondary structure from DSSP in IDR including blanks (idr ss), detailed list of pLDDT scores for the IDR region (idr plddt), idr median plddt, 50 amino acid sequence to the left and to the right of the polyXY center (poly seq left and poly seq right) and their secondary structure annotations (poly ss left, poly ss right). Pipe signs ("|||") indicate the end of the sequence range.

Supplementary table S3: Summary of polyXs and polyXYs and distribution of helical content. Columns indicate: Amino acid type (poly type), count of cases per type (count), percentage over the complete set (perc.), maximum poly size (max size), median poly size (median size), average poly size (mean size), total helix, extended, coil and unfolded content in the poly region by type summing up to 100% (% helix, % extended, % coil, % unfolded). Top polyXs and polyXYs types with the highest helical content are highlighted in green.

Supplementary table S4: Counts of polyXYs by sequence region and distribution of helical content. Columns indicate: amino acid XY (polyXY), count and percentage of polyXYs and fraction of helices in the polyXY region at the N-terminus (count %, % helix), Non Terminus (count %, % helix) and C-Terminus (count %, % helix). The top 3 most frequent polyXYs per sequence region were highlighted in yellow and the top 10 polyXY with the highest helical coverage were highlighted in green.

Supplementary table S5: Enriched GO terms by polyXY with high helical propensity. Columns indicate: Amino acid XY (polyXY type), Gene Ontology identifier (GO id), Gene Ontology term (GO term), count of enriched genes over the total number of genes annotated with the GO term (Overlap), p-value of the enrichment (p-value), adjusted p-value of the enrichment (adj. p-value), odds ratio provided by EnrichR (odds ratio), the combined score provided by EnrichR (combined score), list of enriched genes in set (enriched genes), count of genes that encode proteins with polyXYs with helical content (count genes helix), fraction of these genes compared to the total of enriched genes for the term (fraction genes helix), list of genes followed by an identifier for the polyXY with helical content (enriched genes helix), Gene Ontology ID of the common parent (common parent id) and Gene Ontology term of the common parent (common parent term). Each spreadsheet contains one of the three Gene Ontology aspects: biological process (BP), molecular function (MF) and cellular component (CC).

Supplementary table S6: Enriched GO terms by polyX. Columns indicate: PolyX (poly type), Gene Ontology identifier (GO id), Gene Ontology term (GO term), count of enriched genes over the total number of genes annotated with the GO term (Overlap), p-value of the enrichment (p-value), adjusted p-value of the enrichment (adj. p-value), odds ratio provided by EnrichR (odds ratio), the combined score provided by EnrichR (combined score), list of enriched genes in set (enriched genes), count of genes that encode proteins with polyXs with helical content (count genes helix), fraction of these genes compared to the total of enriched genes for the term (fraction genes helix) and list of genes followed by an identifier for the polyX with helical content (enriched genes helix). Each spreadsheet contains one of the three Gene Ontology aspects: biological process (BP), molecular function (MF) and cellular component (CC).
